# Comparative Analysis of the Thermal Insulation of Traditional and Newly Designed Protective Clothing for Foundry Workers

**DOI:** 10.3390/polym8100348

**Published:** 2016-09-23

**Authors:** Iwona Frydrych, Agnieszka Cichocka, Paulina Gilewicz, Justyna Dominiak

**Affiliations:** 1 Institute of Textile Architecture, Lodz University of Technology, Lodz 90-924, Poland; 800159@edu.p.lodz.pl (P.G.); 140152@edu.p.lodz.pl (J.D.); 2 Central Institute of Labour Protection-National Research Institute, Warsaw 00-701, Poland

**Keywords:** foundry workers, protective clothing, aluminized basalt fabric, aluminized glass fabric, thermal insulation of clothing, underwear

## Abstract

An objective of the undertaken research was checking the applicability of aluminized basalt fabrics for the production of clothing for foundry workers. The results of flammability, the resistance to contact, convective and radiation heat, as well as the resistance to big molten metal splashes confirmed the thesis of applicability of the packages with the use of aluminized basalt fabric content for the assumed purpose; therefore, such protective clothing was produced. Thermal comfort of foundry workers is very important and related to many factors, i.e., the structure of the protective clothing package, the number of layers, their thickness, the distance between the body and appropriate underwear. In the paper, a comparison of the results of thermal insulation measurement of two kinds of protective clothing is presented: the traditional one made of aluminized glass fabrics and the new one made of aluminized basalt fabrics. Measurements of clothing thermal insulation were conducted using a thermal manikin dressed in the protective clothing and three kinds of underwear products covering the upper and lower part of the manikin.

## 1. Introduction

The research project concerns the design process of clothing protecting against flame and heat radiation, containing a new package of materials involving aluminized basalt fabrics and equipped with the shock temperature sensor. The main aim of the undertaken research was producing the protective clothing model for the founder with the use of packages containing aluminized basalt fabrics. So far, such a kind of clothing has been produced with the use of aluminized glass fabrics. These products fulfil their task, but many scientific publications suggest that basalt fibers are characterized by greater values of tensile strength and thermal resistance, because they have a wider range of working temperatures in comparison to the traditionally-used E glass fibers [1,2]. The application temperature of fabrics made of basalt fibers is higher than that of fabrics made of E glass fibers.

In the case of protective clothing, a very important feature influencing its utility is assuring the appropriate protection and comfort in such a way that the job activity is not limited and the dangerous situations causing accidents are not created. An important aspect from the employer point of view is also the product price. The optimization of foundry worker clothing material assembly is still an actuality and takes into consideration first of all new materials produced by new technologies, which are characterized by a high strength and barrier properties, a relatively low mass per square meter and good utility properties at acceptable costs [3].

The analysis of single layer materials [4] indicated two aluminized basalt fabrics of plain and twill weaves, which fulfilled the assumed criteria for the application in protective clothing for the foundry worker. The measurement of their flammability showed that the times of further burning and further glowing were equal to zero. This means that these fabrics are characterized by similar values of mass per square meter and similar values of thermal insulation parameters as appropriate aluminized glass fabrics, which have been applied so far for such clothing.

Following Hrynyk’s and co-workers’ results [4], as well as the conclusion of Hao and Weidong [2], who stated that “aluminized fabrics, acting as an outer thermal reflective layer, could be a component of thermal protective composite clothing”, we decided that the proposed foundry worker clothing assemblies should consist of aluminized basalt fabric and an appropriate lining. We have to make a choice between two aluminized basalt fabrics of different masses per square meter and different weaves (plain and twill), as well as wool or cotton fabrics with flame-retardant finishing, which can be used as the lining. As the reference, we used the assemblies with the aluminized glass fabric content, which have been used so far in such clothing. All proposed assemblies were assessed in the aspect of the resistance to convective, contact and radiative heat, as well as the resistance to big molten metal splashes. The process of assembly selection is described in Section 2.

The thermal comfort of foundry workers is very important and related to many factors, i.e., the structure of the protective clothing package, the number of layers, their thickness, the distance between the body and appropriate underwear. The last part of the research aimed at checking the thermal insulation values of protective clothing sewn from the new material assembly. The purpose of the research presented in Section 3 was a comparative analysis of the thermal insulation of two kinds of protective clothing: the traditional one made with the use of aluminized glass fabrics and new one made with the use of aluminized basalt fabrics. Measurements of clothing thermal insulation were conducted using a thermal manikin dressed in the protective clothing and three kinds of underwear products covering the upper and lower parts of the manikin. The choice of appropriate underwear was an additional task of the research.

## 2. Selection Procedure of Appropriate Assembly for the Foundry Worker Clothing

### 2.1. State of the Art

Using technical textiles and their modifications for the clothing destined for the protection of workers exposed to different types of hazards is especially interesting, because it concerns labor safety and the elimination of risk in the workplace [5,6,7].

Up till now, basalt fabrics were not recognized as a material for application in personal protective equipment; they have been used mostly for technical purposes [8,9,10,11,12,13,14,15]. The results of the preliminary tests carried out at the Central Institute of Labour Protection-National Research Institute on different types of basalt fabrics indicated that these types of fabrics can be used in protective clothing construction. Taking into account the possible intended uses, the most appropriate seemed to be the basalt fabrics finished with aluminum foil, although they passed the contact heat test only at a contact temperature of 100 °C.

The results of preliminary research on the properties of basalt fabrics and their application were described in [2,4,16,17]. Research carried out previously [16,17] aimed at modelling the chosen protective and biophysical properties of clothing assemblies with the use of aluminized basalt content for the application in gloves protecting against thermal and mechanical factors.

Depending on raw materials used in textile assemblies, the final goods can have different resistance values for the particular thermal factors; therefore, the satisfaction of the user can be different. For the protective clothing for users working in hot places, like, for example, the foundry, very important protective properties are: non-flammability, the resistance to contact, convection and radiation heat, as well as the resistance to big molten metal splashes. The higher the efficiency of protection and the wider range of possible applications, the more complex is the clothing construction, which as a consequence raises its manufacturing costs and diminishes work comfort. In this paper, the authors used basalt fabrics as a compromise between manufacturing costs and protective performance levels.

### 2.2. Materials

In Table 1 are presented the basic characteristics of the examined materials, which can be the components of clothing assemblies. The mass per square meter was determined according to the standard EN 12127:1997 [18] and the thickness according to the standard EN ISO 5084:1999 [19].

The basic resistance of the protective clothing should be assured by the non-flammable material or its assembly. The proposed assemblies consisted of two aluminized basalt fabrics (BAZ, BAZ1) of different masses per square meter and different weaves (plain and twill) + wool (WN) or cotton (BN) fabrics with the non-flammable finishing. The results of the resistance to convective, contact and radiation heat, as well as the resistance to liquid metal splashes performed for different kinds of assemblies according to the appropriate standards allow for selecting the best assembly for the mentioned clothing. For comparison purposes, the results obtained for the assemblies with glass fabric (SZ, used so far in such clothing) are also provided.

### 2.3. Measurement Methods Used for the Selection Procedure

For the above-mentioned assemblies, the following tests were performed: flammability, the resistance to contact, convection and radiation heat, as well as the resistance to big molten metal splashes, assessed according to the appropriate standards.

Flammability was measured according to the ISO 15025:2000 [20] procedure. It relies on the exposure of the fabric sample to a small flame coming from a gas blowpipe for 10 s, and then, the times of further burning and glowing were determined. Additionally, the flame spread was observed (i.e., whether the flame reached the top of one of the vertical fabric sample edges) and the creation of burned residues or holes. Flammability measurements were done for four samples (two of them were cut in the warp direction, two in the weft direction) by the edge burning method using the fabric sample mounted in a special frame. There are four efficiency levels depending on the time of further burning and further glowing. For the first efficiency level, the time of further burning should be less than 20 s, for the second, less than 10 s, for the third, less than 3 s, and for the highest, the fourth, the efficiency level of the time of further burning is less than 2 s. The time of further glowing for the first efficiency level is not determined; for the second one, it should be less than 120 s; for the third, less than 25 s; and for the fourth efficiency level, it should be less than 5 s.

The resistance to contact heat of basalt fabrics was assessed according to PN-EN 702:2002 [21]. The sample was placed on the calorimeter and was in contact with the cylinder, which was heated to the temperature of 250 °C, chosen on the basis of the predicted usage conditions. During the measurement, the threshold time is measured (i.e., the time from the first direct flame contact with the heating cylinder to the moment when the temperature increased by about 10 °C). For the resistance to contact heat, the three standard efficiency levels (F1, F2, F3) are given as follows: for F1, the threshold time is in the interval 5–10 s, for F2, 10–15 s, and for F3, times higher than 15 s.

The resistance to convection heat was measured according to PN-EN ISO 367:1996 [22]. The horizontally-placed sample above the gas blowpipe underwent heat flux (of a density of 80 kW/m^2^) arising from the flame. The transmitted heat was measured by the copper calorimeter in direct contact with the sample. The heat transmission index (HTI_24_) is the time for the colorimeter temperature to increase by 24 °C. There are three levels (B1, B2, B3) of efficiency for this property as follows: for B1, the heat transmission index is in the interval 4–10 s, for B2, 10–20 s, and for B3, higher than 20 s.

Resistance to radiation heat was determined according to the procedure described in PN-EN ISO 6942:2005 [23]. The measurement principle relies on the heat radiation of the flux density of 20 kW/m^2^ acting on the fabric specimen in the established time period. The time for the temperature increase by about 24 °C was registered. It is expressed as a radiation transmission heat index t_24_ (RHTI_24_) (s) calculated as the mean from the results of two specimens. There are four efficiency levels (C1, C2, C3, C4) for the radiation heat resistance as follows: for C1, the heat radiation index is in the interval 7–20 s, for C2, 20–50 s, for C3, 50–95 s, and for C4, higher than 95 s.

The resistance to big molten metal splashes was measured according to PN-EN ISO 9185:2009 [24]. Measurement relies on pouring a certain amount of molten metal on the package sample, which is placed in a small frame under the determined angle to the horizontal plane. Above the examined sample, PVC foil was placed. During the measurement, the lowest mass of molten iron that after pouring on the sample causes damage to the PVC foil under the sample is registered. There are three efficiency levels (E1, E2, E3) determining the mass of big metal splashes: for E1, the mass is given in the interval 60–120 g, for E2, 120–200 g, and for E3, higher than 200 g.

### 2.4. Results of Thermal Resistance Measurements and the Final Choice

Concerning the flammability, the measurements were done for single basalt fabrics (not assemblies). In the case of both basalt fabrics, the times of further burning and further glowing were equal to zero; this means they achieved the highest, the fourth, protection efficiency. The obtained results of other measurements are presented in Table 2.

Based on the results from Table 2, it can be stated that the resistance to big molten metal splashes showed that the examined assemblies are at the E3 efficiency level according to the standard PN-EN ISO 11612:2011 [25]. Generally, it can be said that the results obtained for the textile assemblies with the aluminized basalt fabric content are satisfactory, and they showed similar values of resistance as the assemblies with the aluminized glass fabric content for three thermal factors (i.e., the resistance to big metal splashes, to contact heat at 250 °C and to convective heat). In the case of radiation heat, they showed the much higher resistance than the assemblies with the glass fabric content. Additionally, the higher levels of protection efficiency are fulfilled by the assemblies, which contain the thermal insulation lining of WN (wool fabric with the flame-retardant finishing).

On the basis of the results of the performed research, it can be stated that the textile assembly containing the aluminized basalt fabric BAZ of twill weave and a mass per square meter equal to 440 g/m^2^ combined with the flame-retardant wool lining seems to be the best for the protective clothing for foundry workers; therefore, the clothing from such an assembly was sewn and was the object of further measurements of clothing insulation on the thermal manikin.

## 3. Measurement of Clothing Insulation on the Thermal Manikins

### 3.1. State of the Art

Many literature sources assure that thermal manikins are the most often used tools for the clothing thermal insulation measurement [26,27,28,29], because they allow obtaining precise, reliable and repeatable values of measured parameters in the given conditions [27,30]. The number of manikins used for research, as well as the number of their application areas are still growing. Thermal manikins available on the market are characterized by different technical parameters, constructional materials, shape and the number of segments. Very complex manikins are available, consisting of 123 segments with the possibility of sweating [31], as well as more simple models [10] equipped only with the heating function.

Very important is the level of correlation between the results of thermal insulation obtained from measurements on different thermal manikins constructed by different producers. Unfortunately, a great variability of results was stated during the research on the correlation of results carried out on the protective clothing in a few laboratories in 2001 [32]. The reasons were the differences in the construction of the manikins, the different methods of the result calculation and not precise enough control of the measurement conditions. The other round of trials was carried out in three laboratories equipped with three different manikins performed with different clothing types [33]. Two laboratories used the manikin NEWTON consisting of 26 and 34 segments, respectively; whereas the third laboratory had the manikin TORE, consisting of 17 segments. Results for the clothing of lower air permeability showed differences at the level of 6%–15%; whereas, the results for the clothing of higher air permeability showed differences of about 25%–29%. Research carried out for the clothing industry proved that the measurements done on the thermal manikins gave repeatability on the level of variation coefficient equal to 5%.

The difference in the clothing thermal insulation in the climatic chamber without wind [34] carried out on volunteers and the thermal manikin was 13%. A higher thermal insulation was obtained in the case of the measurements on volunteers than on the thermal manikin, but the latter ones were a few times more precise than those carried out on the volunteers (measurement uncertainty for the thermal manikin was 2%; whereas in the case of measurements on volunteers, it was 12%–18%).

Thermal insulation [35] measurements were done for different kinds of clothing, for example for motorcyclist clothing with the lining and membrane, as well as clothing without the lining, but with the membrane. The increase of wind speed during the measurements caused a decrease of the thermal insulation for the clothing with the lining and membrane of about 20%; and 17% for clothing without the lining, but with the membrane.

Measurements of the thermal comfort of soldiers dressed in bulletproof vests and helmets [36] showed different thermal insulation values dependent on the application of the ballistic inserts (hard or soft) and the higher thermal insulation for the body covered more by the vest.

Clothing for surgery [37] has to be resistant to blood and bacteria propagation, and at the same time, it should be characterized by water vapor permeability. In this case, the values of thermal insulation measured on the thermal manikin were higher than the required ones.

The thermal manikin was also used for research done on the vests with phase change materials (PCM) destined for work in moderately hot environments [38]. Two kinds of microcapsules were used in the vests: hexadecane and octadecane. Thermal insulation measured on the thermal manikin was indicated to be the best solution for the vest, into which the mixture of both kinds of capsules was introduced.

Moreover, the thermal manikin was used for the thermal insulation determination of summer and winter Tibetan clothing [39]. It was compared with the clothing sets elaborated by the American Society of Heating Refrigerating and Air conditioning Engineering (ASHRAE), and the Tibetan clothing achieved higher values of thermal insulation of about 0.036 m^2^ K W^−1^.

The results of thermal insulation for the examined clothing sets (jackets filled with duck down and kapok fibers protecting against cold) obtained in East China [40] did not indicate significant differences. Thermal insulation values were confined in the interval of 0.043–0.056 m^2^ K W^−1^.

### 3.2. Materials

**Figure 1:**
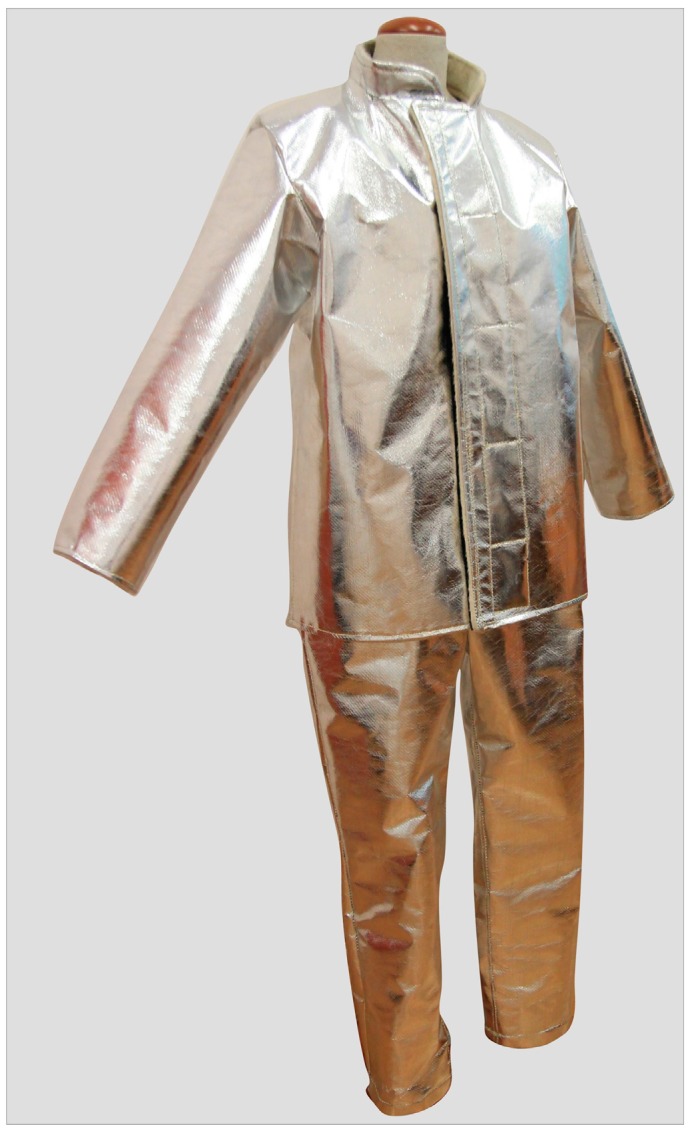
Photo of the clothing for the foundry worker.

Measurements of thermal insulation were performed for the traditional (IZO-TERM, 59-620 Gryfów Śląski, Poland) available on the market protective clothing made with the use of aluminized glass fabric and the new one made with the use of aluminized basalt fabric of the same construction (Figure 1). Additionally, the manikin was first dressed in the underwear covering the upper, as well as the lower body part. The traditional cotton underwear was compared with two innovative underwear products. Clothing sets consisting of protective clothing and appropriate underwear covering the upper and lower parts of the manikin body size (m) were measured on the thermal manikin of size m. Summarizing, the measured objects were two kinds of personalized protective clothing, basalt (BAZ) and glass (SZ), as well as three kinds of underwear products (cotton, B, thermo-active, T, non-flammable finished, N) available on the market, produced by a Polish enterprise (BRUBECK, 98-220 Zduńska Wola, Opiesin, Poland).

U-47-02 (IZO-TERM, 59-620 Gryfów Śląski, Poland) was chosen for the measurement model of protective clothing and was sewn at a Polish company. It consisted of a blouse and trousers (Figure 1). For this version, which was a standard assortment of the company, it contained the aluminized glass fabric ST 97 and thermo-insulation lining made of wool fabric with the flame-retardant finishing 9409/02388 OG.

The blouse consisted of two symmetrical fronts with Velcro and naps. The back was divided into two parts: a yoke was added to the lower part of the back. The sleeves were long and made of one fabric element with the seam placed on the back part of the sleeve.

The trousers consisted of two symmetrical fronts and two symmetrical backs. In the front were placed naps. Additionally, they were equipped with braces without a yoke.

The different types of underwear used for the tests were from the unisex collection. The characteristics of the underwear products are given in Table 3.

The innovation of the underwear products relies, among others, on the application of raw material, which allows for providing the feeling of physiological comfort at a higher level than in the case of traditional cotton underwear. Synthetic fibers used in the innovative multi-layer underwear products allow for air and moisture transportation through the textiles. These solutions are achieved by the selection of appropriate synthetic fibers. The use of different structural parameters of the underwear provides a variety of thermal insulation values.

The special knitwear structure enabled creating separate zones that differ by stitches. The plated stitch contains in its structure two types of yarns, from which underwear goods are produced. The Jacquard stitches include links, which are characterized by low compressibility and provide additional ventilation in areas of potentially increased sweating. Products made with this type of stitch may have a higher air permeability than in the case of a plain knit.

### 3.3. Methodology

Measurements of the thermal insulation of the appropriate sets of protective clothing and underwear were carried out based on the logistic plan given in Table 4 on the thermal manikin PERNIL (placed in the Institute of Textile Architecture of Lodz University of Technology) in the climatic chamber with a temperature of 20 °C and a relative humidity of 43% according to PN-EN ISO 15831:2006 [41].

The model of the thermal manikin was produced by a Danish firm. It consists of 24 segments and has the shape of a woman’s body of size m with a movement option [30,42]. It enabled bending the knees to the angle of 90° and turning up to 100°. Shoulders can be turned in the interval from 0 to 300°, and the head can be rotated to the left and right sides.

According to PN-EN ISO 15831:2006, the total thermal insulation of clothing represents the total thermal insulation, which is between the skin and the surrounding atmosphere, taking into account clothing layers and air layer, measured in the determined conditions with the use of the non-moving thermal manikin. Total thermal insulation was calculated according to the serial model. In the serial model, the determination of thermal insulation is obtained on the basis of weighted areas of particular manikin elements [30,42]. In this model the total thermal insulation is expressed by Equation (1):

(1) $$\mathrm{It}=\sum_{i}f_{i}[\frac{(Tsi-Ta)\cdot ai}{Hci}]\cdot [\frac{m^{2}\cdot k}{W}]$$

where:

It is the total thermal insulation of clothing;
*T*s*i* is the temperature of the shell surface of the *i*-th manikin segment, °C;
*T*a is the air temperature in the climatic chamber, °C;
*H*c*i* is the value of electrical power transmitted through the *i*-th manikin segment, W/m^2^;
*fi* is the coefficient of the area of the *i*-th manikin segment calculated on the basis of Equation (2):
  (2) $$fi=\frac{ai}{A}$$
  where:
*ai* is the area of the *i*-th manikin segment, m^2^;
*A* is the total area of the manikin body.

Covering the manikin body with the underwear and protective clothing took into account the segments presented in Table 5. The head consisted of three segments; hands and feet were not covered by the clothing.

### 3.4. Results

**Figure 2:**
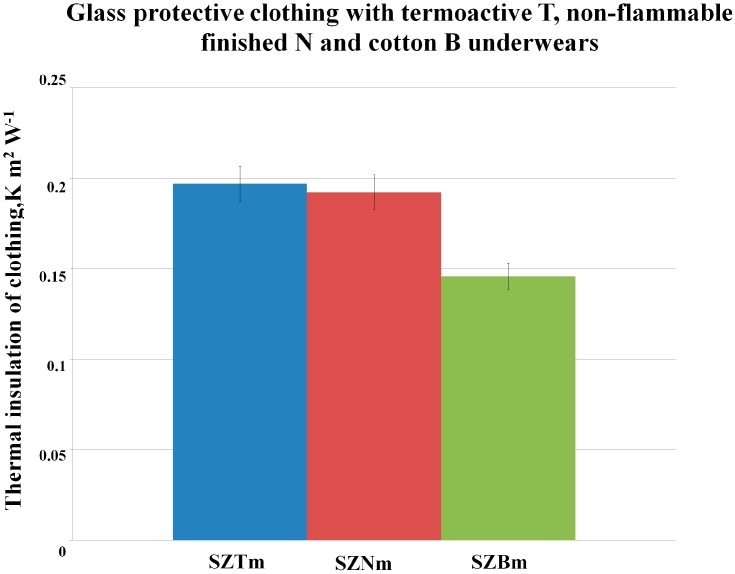
Values of thermal insulation for sets of aluminized glass clothing with three types of underwear products in size m.

**Figure 3:**
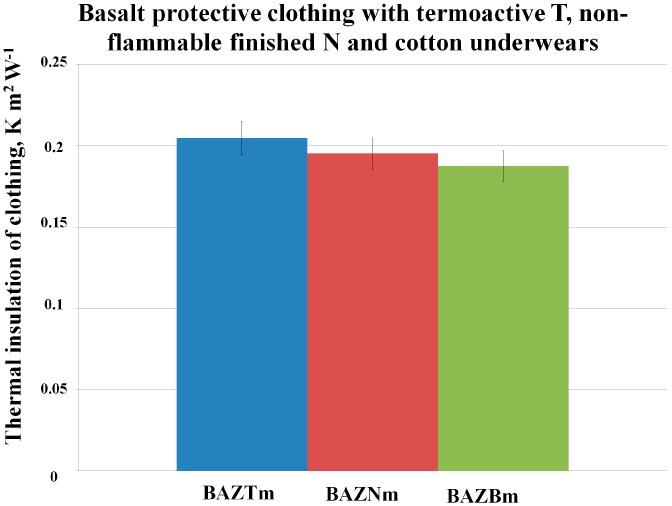
Values of thermal insulation for sets of aluminized basalt clothing with three types of underwear products in size m.

In Figure 2 and Figure 3 are presented successively the results of the thermal insulation of appropriate sets of aluminized clothing (made of glass SZ or basalt BAZ fabrics with the wool lining with the flame-retardant finishing) with three types of underwear: thermo-active, T, non-flammable finished, N, and traditional cotton, B, separately for each kind of protective clothing.

The highest thermal insulation in both cases (for the aluminized glass (SZ) and basalt (BAZ) clothing variants) was observed for the clothing sets with the thermo-active underwear (T).

Comparing the thermal insulation values for sets with the thermo-active and non-flammable finished underwear, it can be noticed that for the aluminized basalt clothing with the underwear in size m (BAZTm, BAZNm), the values are on a similar level as for the aluminized glass clothing with underwear in size m (SZTm).

On the basis of the results from Figure 2 and Figure 3, we can also notice that in all cases, the results of thermal insulation for sets with the clothing made with the use of aluminized basalt fabrics are slightly higher than those for the sets of clothing made with the use of aluminized glass fabrics. The above statement proves that the assumed theory of the replacement of the aluminized glass clothing by the aluminized basalt clothing is justified.

Although the underwear with non-flammable finishing in the sets (SZNm = 0.1922 K m^2^ W^−1^, BAZNm = 0.1953 K m^2^ W^−1^) gave slightly lower thermal insulation results than the thermo-active underwear in the sets (SZTm = 0.1968 K m^2^ W^−1^, BAZTm = 0.2046 K m^2^ W^−1^), its application is justified by this special property. Underwear with non-flammable finishing allows for an increase of the protection against harmful factors, which can appear during work in hot environment conditions; whereas the thermo-active underwear did not provide any protection against flame. Protection of foundry workers is our main aim; therefore, that was the reason that the underwear with non-flammable finishing was assessed as the better one for the foundry worker.

In Figure 2 and Figure 3, it can be observed that in the cases of clothing sets with the traditional underwear products made of cotton (B), the values of thermal insulation are lower (8%) than in the case of the application innovative underwear products.

The processes of heat exchange through the clothing depend mainly on the values of thermal insulation. Research carried out by Holmer [43] showed that the value of thermal insulation of two-part aluminized protective clothing was 1.48 clo (0.2294 K m^2^ W^−1^) The author underlined that it depends on properties of the textile materials used in the clothing, the amount of air in the material structure, as well as between the particular clothing layers. He pointed out that the significant factor influencing the clothing thermal insulation is the amount of air placed between the clothing layers. Research [33] confirms that the air between the clothing layers can improve the thermal insulation of clothing. Often, in order to improve the insulation, the number of clothing layers is increased. Then, the thickness of the clothing product increases, and the bigger amount of air ensures better insulation. Following the last statements, in our further research work, we are going to analyze the influence of different sizes of clothing and underwear on the thermal insulation of such sets.

Summarizing, we can state that the application of the aluminized basalt fabrics combined with the appropriate underwear variant of a good fit allows for obtaining the values of clothing thermal insulation at a similar level or even slightly higher than in the case of commercially-produced clothing made of assemblies with the use of aluminized glass fabrics. This means that the application of less expensive raw materials, i.e., basalt fibers, assures a similar thermal comfort (or better) and functionality for the user.

Carrying out this comparative analysis allows for the selection of potentially the best set of “protective clothing + underwear” on the basis of the results of measurements at the temperature of 20 °C. However, we have to remember that the potential users work in conditions with temperatures higher than 20 °C.

## 4. Summary

The analysis of the protective properties of textile assemblies with the aluminized basalt fabric content showed the same protection efficiency level as the assemblies with the use of aluminized glass fabric content for three thermal factors, i.e., resistance to big molten metal splashes and resistance to contact and convective heat. In the case of radiative heat, the packages with the aluminized basalt fabrics showed much higher resistance than the assemblies with aluminized glass fabrics according to the standard PN-EN ISO 11612:2011. On the basis of the results of the performed research on the protection efficiency levels, it can be stated that the textile assembly containing the aluminized basalt fabric with the twill weave and a mass per square meter equal to 440 g/m^2^ + the wool fabric with the flame-retardant finishing seems to be the best for the protective clothing for foundry workers. This assembly was used for sewing the protective clothing for the foundry worker.

The experiment carried out confirmed that the applied aluminized basalt fabric used in the protective clothing for foundry workers assures at least the same (or slightly better) protection against the thermal factors and the same (or better) thermal comfort at a lower cost as the so far used aluminized glass fabrics.

After the very careful analysis of the measurement results, we recommend using the set of clothing made of aluminized basalt fabric with the non-flammable finished underwear (BAZNm) for the foundry worker, although it provides a slightly lower thermal insulation than the set with the thermo-active underwear (BAZTm), due to the better protection against flame, which occurs in the foundry environment.

The measurements carried out on the thermal manikin for the thermal insulation of clothing, which was personalized for the manikin size, and on the underwear products covering the upper and lower manikin parts showed that using the innovative underwear products made of synthetic fibers is the better solution for foundry workers than the traditional cotton ones.

## Figures and Tables

**Table 1:** Characteristics of the aluminized fabrics and inserts.

Fabric	Aluminized glass fabric ST 97	Cotton fabric with flame-retardant finishing	Wool fabric with flame-retardant finishing	Aluminized basalt fabric	Aluminized basalt fabric
Symbol	SZ	BN	WN	BAZ	BAZ1
Mass per square meter (g/m^2^)	430	250	566	440	319
Thickness (mm)	0.56	0.60	5.31	0.49	0.35
Weave	twill	plain	twill	twill	plain

Where: SZ—aluminized glass fabric; BN—cotton fabric with the flame-retardant finishing; WN—wool fabric with the flame-retardant finishing; BAZ, BAZ1—aluminized basalt fabrics.

**Table 2:** Results of the protection efficiency of thermal factors.

Properties	SZ/BN	SZ/WN	BAZ/BN	BAZ/WN	BAZ1/BN	BAZ1/WN
Resistance to big metal splashes (g)	>220 3 level	>220 3 level	>220 3 level	>220 3 level	>220 3 level	>220 3 level
Resistance to contact heat at 250 °C	7.4 s 1 level	16.4 s 3 level	8.0 s 1 level	17.0 s 1 level	7.8 s 1 level	16.4 s 3 level
Resistance to convective heat HTI_(24)_ (s)	9.3 1 level	13.5 2 level	7.4 1 level	13.6 2 level	7.1 1 level	13.4 2 level
Resistance to radiation heat RHTI_(24)_ (s)	41.9 2 level	78.7 3 level	150.3 4 level	278.9 4 level	184.7 4 level	235.7 4 level

**Table 3:** Characteristics of underwear products.

Kind of underwear	Symbol of underwear	Stitch	Mass per square meter (g/m^2^)	Thickness (mm)	Lk/1 (cm)	Lr/1 (cm)	Raw material content
Cotton	B	Plain	220	0.81	10	17	100% cotton
Thermo-active	T	Plated plain with jacquard elements	155	0.70	16	18	54% PA (polyamide), 44% PES (polyester), 2% Elastane
Non-flammable finished	N	Plated plain	158	0.71	15	22	54% Modal Protect, 27% cotton, 19% PA (polyamide)

Where: Lk—wale count/1 cm; Lr—course count/1 cm.

**Table 4:** Sets of clothing protecting against flame and heat radiation for the measurement on the thermal manikin of the (m) medium size.

Kind of underwear	Symbol of underwear	Set of clothing
Cotton	B	Basalt (BAZm)
Thermo-active	T	Basalt (BAZm)
Flame-retardant	N	Basalt (BAZm)
Cotton	B	Glass (SZm)
Thermo-active	T	Glass (SZm)
Flame-retardant	N	Glass (SZm)

Where: BAZm—basalt fabric with the underwear of medium size (m); SZm—glass fabric with the underwear of medium size (m).

**Table 5:** Segments of the manikin used for the measurement.

Number of segments	Name of segment	Area of segment (m^2^)
1	Left Low leg	0.0975
2	Right Low leg	0.0975
3	Left Front Thigh	0.0858
4	Right Front Thigh	0.0858
5	Left Back Thigh	0.0858
6	Left Back Thigh	0.0858
7	Pelvis	0.0558
8	Left Back side	0.0408
9	Right Back side	0.0408
10	Left Forearm	0.05
11	Right Forearm	0.05
12	Left Upper arm	0.073
13	Right Upper arm	0.078
14	Left Chest	0.07
15	Right Chest	0.07
16	Left Back	0.065
17	Right back	0.065
